# Causes of Mortality in Intensive Care Units for Patients with Chronic Inflammatory Diseases from the French National Health Data System

**DOI:** 10.3390/jcm14062000

**Published:** 2025-03-15

**Authors:** Yannis Hamidou, Jean Marc Sobhy Danial, Thibault Balcaen, Sophie Liabeuf, Solène Laville, Claire Jesson, Pierre Antoine Bruy, Camille Farnos, Marie Doussiere, Vincent Goeb

**Affiliations:** 1 Department of Rheumatology, University Hospital of Amiens, 80054 Amiens, France; sobhydanial.jean-marc@chu-amiens.fr (J.M.S.D.); jesson.claire@chu-amiens.fr (C.J.); bruy.pierreantoine@chu-amiens.fr (P.A.B.); farnos.camille@chu-amiens.fr (C.F.); doussiere.marie@chu-amiens.fr (M.D.); goeb.vincent@chu-amiens.fr (V.G.); 2 Department of Medical Information, University Hospital of Amiens, 80054 Amiens, France; balcaen.thibault@chu-amiens.fr; 3 Department of Clinical Pharmacology, University Hospital of Amiens, 80054 Amiens, France; liabeuf.sophie@chu-amiens.fr (S.L.); laville.solene@chu-amiens.fr (S.L.)

**Keywords:** inflammatory disease, biological treatment, conventional treatment, mortality, intensive care, reanimation, cardiovascular, French national health database

## Abstract

**Background/Objectives**: Inflammatory pathologies are at the center of various medical specialties and benefit from conventional treatments as well as biological treatments. These latter ones have often been the subject of studies yielding heterogeneous results regarding their infectious and mortality risks. This work aims to describe mortality and its causes in patients afflicted by inflammatory pathologies, receiving either conventional or biological therapy during their first stay in intensive care units. **Methods**: Our study was conducted using the French national health database, encompassing all hospital stays on a national scale. All comparisons between conventional treatment and biological therapies were performed using the Chi-square test, Fisher’s exact test, or Student’s *t*-test. **Results**: In total, 13,816 patients were included. Within 90 days of the first admission to the intensive care/reanimation service, 11.6% of the patients died, including 9.4% within 30 days and 7.3% during hospitalization. More patients died in the conventional treatment group in comparison to the biological treatment group. More deaths were observed due to cardiovascular (27%), infectious (15%), gastroenterological (12%), and oncological (12%) conditions in the conventional treatment group. However, there were as many deaths from oncological causes (19%) as from cardiovascular causes (19%) in the biological therapy group. Hypertension (66.8%) and renal insufficiency (50.4%) were the most frequently associated comorbidities with mortality. **Conclusions**: Mortality in intensive care/reanimation during the initial stay of patients afflicted by inflammatory pathologies is of greater concern for those treated with conventional treatments. Causes of death tend to be more cardiovascular and require more prevention and care management.

## 1. Introduction

The treatment of chronic inflammatory diseases has been revolutionized by the advent of biomedicines, in particular immunotherapies targeting different inflammatory mediators or mechanisms (TNF, IL6, CTLA4, JAKi, etc.) depending on their relevance to the pathophysiology of the disease being treated (rheumatoid arthritis, axial spondylarthritis, inflammatory bowel disease, psoriasis, etc.) [1,2,3,4,5].

These treatments are used in the case of failure or intolerance to conventional disease-modifying therapies and offer greater therapeutic efficacy. However, poor tolerability may be the price to pay for their use, with a sometimes significant risk of infectious, cardiovascular, or even neoplastic side effects [6,7,8]. In the literature, many safety studies of conventional background treatments or biologicals have been conducted based on each therapeutic class studied in isolation, and there are few studies comparing treatments on a large scale [9,10,11] or under conditions of daily use. Some studies have compared the safety profile of certain biologicals in real-life settings, but the numbers involved are limited, and the results are inconsistent and sometimes biased [12,13,14,15].

National cohorts have been set up in some countries, but they focus on a specific disease [16] and do not necessarily have the resources to extend to all existing biomedicines and inflammatory diseases being treated. The aim of our work is thus to describe at a national level, here of the French State, the causes of mortality in patients with chronic inflammatory diseases treated with synthetic conventional therapy (SCT) and/or biologicals at the time of their admission to an intensive care unit, using an official database.

## 2. Materials and Methods

### 2.1. Treatments of Interest for the Study

This retrospective descriptive study was based on the Programme de Médicalisation des Systèmes d’Information—PMSI (Medicalization of Information Systems Program), an IT tool providing a standardized description of medical activities in healthcare institutions, and the Système National des Données de Santé—SNDS (National Health Data System). Patients were defined from these databases using the International Classification of Diseases (ICD-10) coding system. The treatments dispensed to patients were identified using the Dispensing Unit Code (UCD) coding system for drugs used in hospitals and the Presentation Identifier Code (CIP) coding system for drugs dispensed in the community. The study covers a wide range of inflammatory diseases for which biologicals are indicated: Crohn’s disease, ulcerative colitis, psoriasis, lupus erythematosus, Still’s disease, giant cell arteritis, rheumatoid arthritis, spondylarthritis, juvenile arthritis and arthropathy in Crohn’s disease.

Among the SCT, we included methotrexate (MTX), leflunomide, sulfasalazine, hydroxychloroquine, mycophenolate mofetil, azathioprine, and cyclophosphamide. The latter were included using data on dispensing in cities via their CIP codes. Biologics offer healthcare professionals ideal therapeutic alternatives. The following biologicals were selected for this study: TNF-i (infliximab, adalimumab, etanercept, certolizumab, golimumab), ustekinumab, secukinumab, ixekizumab, sarilumab, tocilizumab, abatacept, rituximab and anakinra. Finally, this section also includes two JAK inhibitors: upadacitinib and baricitinib.

### 2.2. Patient Selection Algorithm

**Figure 1:**
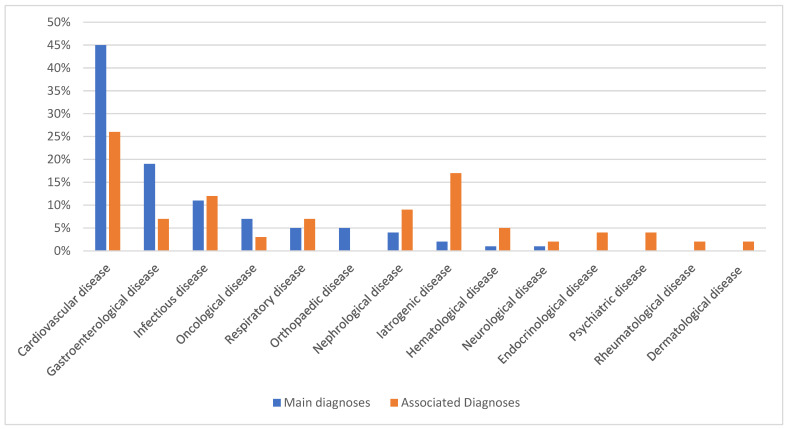
Distribution of the 100 most frequent main diagnoses and associated diagnoses in intensive care units.

To be included, patients had to have had (i) at least one hospitalization or long-term health condition with a diagnosis of inflammatory disease coded as such between 2012 and 29 February 2020, (ii) at least one administration of SCT or biological after the first diagnosis of inflammatory disease between 2015 and 29 February 2020 (inclusion index date), and (iii) a stay in an intensive care unit after the inclusion date (Figure 1). 

Exclusion criteria were age less than 17 years and first treatment before the date of first inflammatory disease diagnosis. The selection dates of inflammatory disease diagnoses were chosen to include only incident cases from the first administration of the index treatment (corresponding to the inclusion date).

For all these patients, we identified all drug deliveries of candidate treatments (SCT or biological) in hospital or in the community, as well as treatments with corticoids or nonsteroidal anti-inflammatory drugs delivered in the last 15 months preceding the stay in intensive care. For all these patients, we looked for the presence of comorbidities identified during a previous hospitalization or in the reasons for long-term health conditions. Only comorbidities diagnosed before the first admission to the intensive care unit were included. For all these patients, we identified deaths during the stay and 30 and 90 days after discharge. Causes of death were also identified, if available, via the database of the Centre d’épidémiologie sur les causes médicales de décès—CepiDC (Center for Epidemiology of Medical Causes of Death).

### 2.3. Statistical Analysis

Statistical analyses were performed using R software version 4.3.1. All univariate analyses were performed according to a binomial distribution. Analyses between two categorical variables were interpreted using the Chi 2 test or the Fisher test. Analysis of variance (ANOVA) tests were used when a qualitative variable was compared with a quantitative variable.

### 2.4. Ethics and Data Availability

The data generated by this study are not available to the general public.

The Amiens Picardie University Hospital has permanent access to the SNDS data collected in accordance with Decree No. 2021-848 of 29 June 2021 on the processing of personal data.

CHU Amiens Picardie keeps an internal register of persons trained in the use of data who have a right of access to the SNDS. It also keeps an internal register of all work involving the use of these data in accordance with the security guidelines of the SNDS. As all data are anonymous, informed consent was not required.

## 3. Results

### 3.1. Population Characteristics

**Figure 2:**
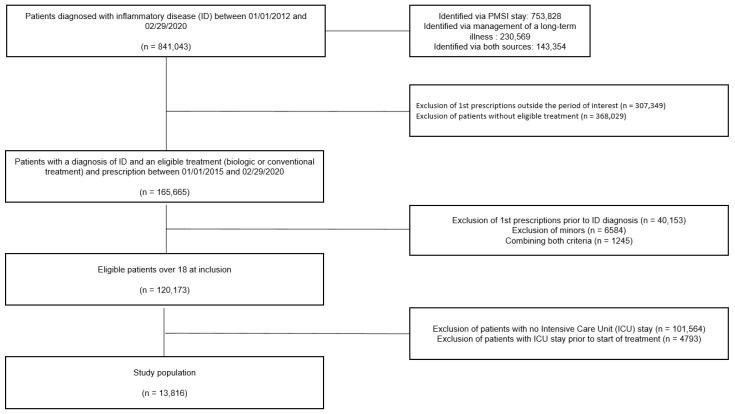
Flow chart.

A total of 13,816 patients were analyzed (Figure 2).

The median age of the patients was 63 years [50–73], and 53% were men. The most common comorbidities were hypertension (68.4%), type 2 diabetes (28.8%), and chronic renal failure (29.3%) (Table 1).

The majority of patients were treated with conventional therapy, with 19.9% of patients receiving biological treatment as monotherapy (Table A1 and Table A2 in Appendix A). The main indications for treatment were inflammatory rheumatological, gastrointestinal, and dermatological diseases. At the time of enrolment, 81.6% of patients were receiving SCT, with the majority receiving MTX (63.3%). Of the patients receiving biological, 15.7% were treated with TNF-i (Table A1 and Table A2 in Appendix A). The median time from indication to first referral was 8 months. The median time from enrolment to ICU admission was 24 months.

### 3.2. Patient Characteristics During ICU Stays

**Figure 3:**
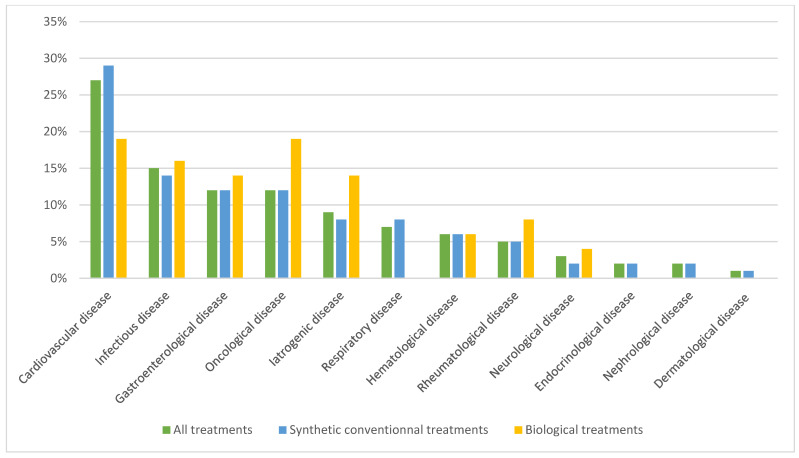
Causes of death according to CepiDC.

The majority of principal diagnoses associated with ICU stays are related to cardiovascular disease (Figure 3). Of the associated diagnoses, 26% are of cardiovascular origin, and 17% are related to iatrogenic conditions (Figure 3). In the 90 days following discharge from the ICU, 1607 patients died. Of these, 1304 died within 30 days of ICU discharge, of whom 1014 died during hospitalization.

### 3.3. Comparison of Populations According to Treatment Prior to Admission to the Intensive Care Unit

Between the two groups, patients treated with biologicals were younger, more likely to be male, and had fewer comorbidities. The indications for biological treatments were mainly gastroenterological with a j. Psoriasis was also represented in both groups. There was an over-representation of spondylarthritis patients in the biological treatment group compared with the SCT group. The opposite is true for patients with Horton’s disease or RA. The proportion of deaths within 90 days of ICU discharge was higher in the SCT group (12.9%) than in the biological group (7.9%). The tendency was identical for deaths within 30 days of discharge and during hospitalization, with more deaths in the SCT group (Table 2). Of the patients who died prior to ICU admission, the majority were men with a median age of 73 years. The last treatment before ICU admission in the deceased population was more often SCT. In addition, 73.9% and 61.7% of patients who died had received corticosteroid or nonsteroidal anti-inflammatory drug therapy, respectively, in the 15 months before ICU admission. Finally, RA and psoriasis were the most common diseases among patients who died (Table 3).

### 3.4. Analysis of Causes of Death (n = 361)

Analysis of the causes of death was carried out using the CepiDC databases for 361 of the 1014 patients selected (lack of data due to incomplete collection from the database). The four main causes of death were cardiovascular, infectious, gastroenterological, and oncological.

In the SCT group, 312 patients died. The main causes of death were also cardiovascular, infectious, and gastroenterological. In the biological treatment group, 49 patients died, with oncological, cardiovascular, and infectious causes predominating.

## 4. Discussion

We described the mortality of patients with chronic inflammatory diseases according to the type of treatment received at the time of their first intensive care unit (ICU)/resuscitation stay using a national database.

Of the 13,816 patients included, the main reasons for admission were cardiovascular and gastroenterological, accounting for 45% and 19%, respectively. The statistics for organ failure in intensive care units in the general French population show that respiratory (70.3%) and cardiovascular (51.3%) disorders are more common, while gastroenterological disorders are in fifth place with 3.6% [17]. Treatment of chronic inflammatory diseases, therefore, does not appear to have a predominant influence on the risk of cardiovascular disease. Chronic inflammatory diseases alone carry an inherent cardiovascular risk [18] that is higher than that of the general population [19].

The over-representation of acute gastroenterological conditions treated included inflammatory enteritis or ulcerative colitis (6.2%), bowel obstruction (1.8%), and diverticulosis (1%). It should be noted that more than ¾ of our patients were receiving corticosteroid therapy. Regardless of the treatment prescribed, the management of patients with chronic inflammatory diseases must focus on monitoring for the occurrence of this type of gastroenterological complication. Of all selected patients, more than ¾ were receiving SCT (74.3% at the time of their ICU admission), which is consistent with good clinical practice for first-line treatment according to various international professional societies, regardless of the indication [20,21,22,23]. Biologics and JAK inhibitors remain second-line treatment options in the event of ineffectiveness or intolerance to SCT. TNF-i are the most widely used, as they have multiple cross-disciplinary indications [24]. In addition to their cost, this position as second-line drugs has been justified in practice by fears of an increased risk of adverse effects. In this real-life study, it was found that the exposure to biological treatments in patients who were hospitalized and died in intensive care was actually significantly lower than in the SCT group. Older patients with more comorbidities are less eligible for biological treatments in current practice. In fact, there were 5.1% deaths during hospitalization in the biologicals group compared to 8.1% in the SCT group, 6.7% deaths 30 days after hospital discharge in the biologicals group compared to 10.4% in the SCT group, and 7.9% deaths 90 days after hospital discharge in the biologicals group compared to 12.9% in the SCT group.

As a result, our data provide a reassuring tendency that patients exposed to biological treatments are not at higher risk than those exposed to SCT monotherapy. This may be explained by the fact that the underlying disease is better controlled with the use of a biological, and therefore, the deleterious role of inflammation in the occurrence of an adverse effect is reduced. In addition, patients in the synthetic treatment group have more comorbidities, are older, and therefore at greater risk of death. Regardless of the treatment on admission of an ICU patient with inflammatory disease, mortality rates were lower than the overall ICU mortality rate in the general population, estimated to be around 17.5% between 2013 and 2019 [17]. The most common causes of death were cardiovascular (26%), infectious (15%), gastroenterological (12%), and oncological (12%), all treatments of interest combined. Although infectious causes of admission were less common (11%), they appear to be more important when the causes of death of patients are counted. According to the CepiDC databases, the top 3 causes of death in the general population between 2015 and 2020 were oncological (29.7%), cardiovascular (22.5%) and respiratory (7%) [25]. This could explain the higher number of deaths in the synthetic treatment group, as these causes can sometimes be a contraindication to biological treatments. Patients with chronic inflammatory diseases are, therefore, at greater risk of dying from infectious causes than the general population, irrespective of the treatment they receive. This has already been clearly described for corticosteroid therapy alone [26,27] and is therefore also observed with disease-modifying therapies, although our data do not allow us to distinguish the responsibility of one type of treatment from the other. In the absence of individualized data, it is not possible to determine the doses of corticosteroids given to patients, as the threshold of 10 mg daily has been shown to be associated with a significant excess risk of infection. Deaths in SCT were more likely to be due to cardiovascular (29%), infectious (14%), gastroenterological (12%), or oncological (12%) causes, whereas oncological (19%), cardiovascular (19%) or infectious (16%) causes were more common in patients treated with biologicals.

In clinical practice, the emphasis is on prevention and regular assessment of oncological and infectious risk during initiation and follow-up of patients treated with biologicals [28]. The latter seems justified, given the causes of death in the biological group. However, one of the most important lessons to be learned from this study is that cardiovascular mortality is predominant, regardless of the treatment received, and there are currently few recommendations for managing this specific risk in current practice. This cardiovascular risk may be observed in inflammatory diseases considered to be not very active [29]. It has recently been highlighted by the re-evaluation of the possibility of prescribing JAKi [30,31].

The median time from enrolment to ICU/resuscitation was approximately 2 years. This can be considered rather short, with a short exposure time that can lead to major complications. The time from indication to the first administration of the treatment of interest is approximately 8 months, which in clinical practice may correspond to the time required to perform all the additional tests and investigations needed to support the various diagnoses. However, this timeframe is not compatible with the ’treat to target’ therapeutic approach that is central to the management of certain diseases (e.g., Rheumatoid arthritis).

Particular attention must, therefore, be paid to cardiovascular and infectious risks, especially during the first two years of treatment, whether SCT or biological.

The strengths of this study lie in the large number of patients analyzed. The cross-sectional approach with several chronic inflammatory diseases in different specialties is not only a strength but also an original feature of this study. Mortality was chosen as the primary endpoint, so the differences highlighted have a relevant clinical impact.

The limitations of this study lie in its descriptive nature. Biologic treatments are of interest to patients whose pathology appears to be more severe and are more often used for gastroenterological diseases. The latter exposes patients to a higher risk of infectious and sometimes postoperative complications, making them inherently more at risk of death. In addition, patient characteristics are not homogeneous, with more comorbidities in the group of patients treated with SCT. Our study does not provide data on mortality rates prior to ICU admission or following the conclusion of the follow-up period.

Patients receiving biologicals may have encountered fatal incidents outside the hospital.

Unfortunately, the health database used does not allow us to obtain individualized data for each patient. As a result, there is a lack of data on the activity and severity of pathologies, patient history as well as on the chronology of the various treatments received by patients. It was also not possible to identify all criteria for patient frailty. Those in the SCT group were older and had more comorbidities. 

On the basis of this database, it seems highly appropriate to continue the work with stratification according to the type of pathology but also the type of biological received by the patients.

A number of biases can be identified in this study. Firstly, the transition to intensive care/resuscitation subjects patients to a selection process in which they are more fragile and at greater risk of death than the rest of the general population suffering from the same pathologies. Those on biologicals may have had a lower likelihood of ICU admission but a higher risk of dying before hospitalization. There may also be selection bias due to the use of administrative databases used to assess reimbursement for care. In fact, some patients may have been excluded if treatment was first provided before a condition was declared to be a long-term health condition. Finally, classification bias may have occurred due to errors in the coding of the different pathologies or comorbidities of interest.

## 5. Conclusions

SCTs and biologicals constitute a therapeutic arsenal used in current practice for the management of certain chronic inflammatory diseases.

Using the National Health Data System database, this study revealed a higher proportion of deaths in patients treated with conventional therapies. The main causes of death were cardiovascular, infectious, gastroenterological, and oncological, and corresponded to the main reasons for admission to intensive care units. Assessment and prevention of cardiovascular risk, therefore, appear to be of vital importance in the management of patients with chronic inflammatory diseases.

However, the results must be weighed against the limitations of this study and the impossibility of making a clinically relevant comparison between the groups. In fact, the variability of the clinical profiles and the intrinsic risks associated with the diseases suggest that a disease-specific analysis would be more appropriate and provide better comparability between the groups.

Further work on the database, focusing on clinically comparable populations, could provide additional information to optimize therapeutic choices. The main goal remains to improve the personalization of treatments.

## Figures and Tables

**Table 1:** Characteristics of patients in the total population.

Variables	Total Population (*n* = 13,816)
Patient characteristics	
Age at inclusion (years)	63 [50–73]
Age at ICU admission (years)	65 [52–75]
Gender Male	7307 (52.9%)
Comorbidities	
Hypertension	7381 (68.4%)
Obesity	3839 (35.6%)
Chronic Kidney Disease	3163 (29.3%)
Type 1 diabetes	841 (7.8%)
Type 2 diabetes	3110 (28.8%)
Cancer	3078 (28.5%)
Congestive Heart Failure	2917 (27.0%)
COPD	1963 (18.2%)
Treatment	
Conventional treatment	11,014 (79.7%)
Biologic	2746 (19.9%)
tsDMARD	6 (<0.1%)
Combination of biological and conventional treatment	50 (0.4%)
Indication for treatment (first indication)	
Rheumatoid arthritis	2831 (20.5%)
Crohn’s disease	2525 (18.3%)
Ulcerative colitis	2439 (17.7%)
Psoriasis	2116 (15.3%)
Spondyloarthritis	1842 (13.3%)
Giant cell arteritis	816 (5.9%)
Lupus	318 (2.3%)
Still’s disease	87 (0.6%)
Juvenile idiopathic arthritis	10 (<0.1%)
Arthropathy in Crohn’s disease	1 (<0.1%)
Combiantion of indications	831 (6.1%)

Continuous variables are presented as median [Q1–Q3]. Categorical variables are presented as *n* (%). COPD: Chronic Obstructive Pulmonary Disease.

**Table 2:** Patient characteristics by type of last treatment prior to ICU stay.

Variables	Biologic Treatment (*n*= 3480)	Conventional Treatment (*n*= 10,265)	*p* Value
Patient characteristics			
Age at ICU admission (years)	56 [42–67]	68 [56–77]	*
Gender Male	2121 (61.0%)	5145 (50.1%)	*
Comorbidities			
Hypertension	1263 (36.3%)	5807 (56.6%)	*
Obesity	867 (25%)	2777 (27.1%)	*
Chronic Kidney Disease	517 (14.9%)	2504 (24.4%)	*
Type 1 diabetes	142 (4.1%)	662 (6.5%)	*
Type 2 diabetes	584 (16.8%)	2374 (23.1%)	*
Cancer	529 (15.2%)	2437 (23.7%)	*
Congestive Heart Failure	433 (12.4%)	2377 (23.2%)	*
COPD	335 (9.6%)	1551 (15.1%)	*
Inflammatory disease			
Rheumatoid arthritis	209 (6.0%)	2600 (25.3%)	*
Crohn’s disease	1072 (30.8%)	1450 (14.1%)	*
Ulcerative colitis	548 (15.7%)	1867 (18.2%)	*
Psoriasis	558 (16.0%)	1555 (15.2%)1	0.21
Spondyloarthritis	819 (23.5%)	1009 (9.8%)	*
Giant cell arteritis	55 (1.6%)	760 (7.4%)	*
Lupus	4 (0.1%)	314 (3.1%)	*
Juvenile idiopathic arthritis	4 (0.1%)	6 (<0.1%)	0.28
Still’s disease	36 (1.0%)	51 (<0.1%)	*
Death			
Death à +/− 90 days	275 (7.9%)	1324 (12.9%)	*
Death à +/− 30 days	233 (6.7%)	1068 (10.4%)	*
Death during stay	178 (5.1%)	832 (8.1%)	*

Continuous variables are presented in median [Q1–Q3]. Categorical variables are presented in *n* (%). * The difference between treatment groups is statistically significant (*p* < 0.05). COPD: Chronic Obstructive Pulmonary Disease.

**Table 3:** Patient characteristics according to vital status at hospital discharge.

Variables	Deceased Patients (*n* = 1014)	Survivors Patients (*n* = 12,802)	*p* Value
Patient characteristics			
Age at inclusion (years)	71 [61–80]	62 [49–73]	*
Age at ICU admission (years)	73 [63–82]	64 [51–75]	*
Gender Male	579 (57.1%)	6734 (52.6%)	*
Last treatment before stay			
Conventional treatment	834 (82.2%)	9431 (73.7%)	*
Biologic treatment	176 (17.4%)	3304 (25.8%)	*
Comorbidities			
Hypertension	677 (66.8%)	6704 (52.4%)	*
Obesity	283 (27.9%)	3556 (27.8%)	0.93
Chronic Kidney Disease	511 (50.4%)	2652 (20.7%)	*
Type 1 diabetes	82 (8.1%)	759 (5.9%)	*
Type 2 diabetes	304 (30.0%)	2806 (21.9%)	*
Cancer	334 (32.9 %)	2744 (21.4%)	*
Congestive Heart Failure	359 (35.4%)	2558 (20.0%)	*
COPD	215 (21.2%)	1748 (13.7%)	*
Treatment indication (excluding multiple indications)			
Rheumatoid arthritis	301 (29.7%)	2530 (19.8%)	*
Crohn’s disease	111 (11.0%)	2414 (18.9%)	*
Ulcerative colitis	127 (12.5%)	2312 (18.1%)	*
Psoriasis	207 (20.4%)	1909 (14.9%)	*
Spondyloarthritis	87 (8.6%)	1755 (13.7%)	*
Giant cell arteritis	97 (9.6%)	719 (5.6%)	*
Lupus	26 (2.6%)	292 (2.3%)	0.56
Still’s disease	11 (1.1%)	76 (0.1%)	0.06
Juvenile idiopathic arthritis	0 (0%)	10 (<0.1%)	1
Arthropathy in Crohn’s disease	0 (0%)	1 (<0.1%)	1
Other treatments given in the last 15 months			
Oral corticosteroids	749 (7.9%)	9583 (74.9%)	0.49
Nonsteroidal anti-inflammatory drugs	626 (61.7%)	8248 (64.4%)	0.09

Continuous variables are presented as median [Q1–Q3]. Categorical variables are presented as *n* (%). * The difference between treatment groups is statistically significant (*p* < 0.05). COPD: Chronic obstructive pulmonary disease.

## Data Availability

Data are unavailable due to privacy.

## Abbreviations

The following abbreviations are used in this manuscript:

CEPI-DC	Center for Epidemiology of Medical Causes of Death
CIP	Presentation Identifier Code
COPD	Chronic Obstructive Pulmonary Disease
CTLA-4	Cytotoxic T-lymphocyte-associated antigen 4
ICU	Intensive Care Unit
ID	Inflammatory Disease
IL-6	Interleukin 6
JAKi	Janus Kinase Inhibitor
MTX	Methotrexate
PMSI	Medicalization of Information Systems Program
RA	Rheumatoid Arthritis
SCT	Synthetic conventional therapy
TNF	Tumor Necrosis Factor
TNF-i	Tumor Necrosis Factor Inhibitor
tsDMARD	targeted synthetic disease-modifying antirheumatic drug
UCD	Dispensing Unit Code

## Appendix A

**Table A1:** Distribution of conventional treatments (*n*= 11,258).

Variables	Patients Numbers
Conventional treatment	
Methotrexate	8749 (63.3%)
Azathioprine	1364 (9.9%)
Mycophenolate Mofetil	362 (2.6%)
Sulfasalazine	284 (2.1%)
Apremilast	243 (1.8%)
Leflunomide	173 (1.3%)
Cyclophosphamide	52 (0.4%)
Chloroquine	21 (0.2%)
Combination of conventional treatments	
Methotrexate; Mycophenolate mofetil	4 (<0.1%)
Azathioprine; Methotrexate	3 (<0.1%)
Methotrexate; Sulfasalazine	2 (<0.1%)
Apremilast; Methotrexate	1 (<0.1%)

Categorical variables are presented as *n* (%).

**Table A2:** Distribution of biological treatment (*n* = 2558).

Variables	Patients Numbers
Anti-TNF α	2814 (15.7%)
Adalimumab	1428 (10.3%)
Etanercept	305 (2.2%)
Infliximab	225 (1.6%)
Golimumab	179 (1.3%)
Certolizumab pegol	47 (0.3%)
Anti-IL12/23	
Ustekinumab	101 (0.7%)
Anti-IL17	76 (0.6%)
Secukinumab	69 (0.5%)
Ixekizumab	7 (0.1%)
Anti-IL1	
Anakinra	56 (0.4%)
Anti-IL6R	55 (0.4%)
Tocilizumab	54 (0.4%)
Sarilumab	1 (<0.1%)
Anti-CD20	
Rituximab	21 (0.2%)
Ig-CTLA-4	
Abatacept	10 (0.1%)
Inhibiteur de JAK (tsDMARD)	6 (<0.1%)
Tofacitinib	5 (<0.1%)
Baricitinib	1 (<0.1%)
Anti-TNF α; traitement conventionnels	47 (0.3%)
Adalimumab; Azathioprine	30 (0.2%)
Adalimumab; Methotrexate	6 (<0.1%)
Infliximab; Azathioprine	6 (<0.1%)
Etanercept; Methotrexate	4 (<0.1%)
Golimumab; Sulfasalazine	1 (<0.1%)
Ig-CTLA-4; traitement conventionnels	
Abatacept; Methotrexate	1 (<0.1%)
Anti-IL6R; traitements conventionnels	
Tocilizumab; Methotrexate	1 (<0.1%)

Categorical variables are presented as *n* (%). TNF: Tumor Necrosis Factor. IL: Interleukine. CD: Differenciation cluster. Anti-IL6R: Anti-IL6 Receptor. CTLA-4: Cytotoxic T-lymphocyte associated antigen 4. tsDMARD: targeted synthetic disease-modifying antirheumatic drug.
